# Supplementary material for: Beta decoupling relationship between CO2 emissions by GDP, energy consumption, electricity production, value-added industries, and population in China
Source: PLoS One. 2021 Apr 1;16(4):e0249444. doi: 10.1371/journal.pone.0249444 (PMC8016343; doi:10.1371/journal.pone.0249444)
Supplement: S1 File — (DOCX) [file pone.0249444.s001.docx]

## Beta decoupling relationship between CO2 emissions by GDP, energy consumption, electricity production, value-added industries, and population in China

Rabnawaz Khan^1*^

^1*^School of Finance and Economics, Fujian University of Technology, No. 33 Xueyuan Road, University Town, Minhou, Fuzhou City, Fujian Province, China

[khan.rab@stmail.ujs.edu.cn](mailto:khan.rab@stmail.ujs.edu.cn)

*Corresponding Author: [khan.rab@stmail.ujs.edu.cn](mailto:khan.rab@stmail.ujs.edu.cn)

## Acknowledgement

I really acknowledge the helpful instruction and comments from my supervisor and admiringly acknowledge the financial support of the National Natural Science Foundation of China of Fujian University of Technology. I exceedingly appreciate my parents and family who encourage me to as a scholar and I cannot ignore the value of the poor student who scarifies all emotions and sentiments for their bright future. In last I really recognize to be-love supporter who hold me and give me strength and power for extant and enduring.

## Abstract

## The sources of fossil energy are the foundation of economic growth due to its aptitude to influence security, socio-economic stability, and the environment. As the fossil energy sources supply has become progressively stern, reconnoitering the beta decoupling relationships between CO2 emissions, GDP, energy consumption, electricity consumption, value-added industries, and population in China, and the result will be favorable for illustrative the security of the resources. This paper uses the environmentally extended stochastic model (STIRPAT), with Beta Decoupling Techniques (BDT), which is using to measure decoupling situation by alpha and beta effects in the period of 1989 to 2018, to calculate % change in CO2 emissions by GDP and energy consumption. The results are strongly negative, and economic growth increasingly depends on coal and natural gas (1) The CO2 emissions annually increasing cause of rapid growth, energy consumption, and generation of electricity, and the structural contradiction of energy safely remained static. (2) Value-added industries estimated that CO2 emissions reduce by primary industries. (3) The decoupling states of CO2 emissions and population show the inverse relationship. This paper recommends that China is sustainable and strengthen energy output, effectively transmute the structure of energy consumption, and promote development policies under the environmental circumstance.

## Keywords: Beta decoupling Technique, STIRPAT, economic growth, energy consumption, electricity production, value-added industries, China 1. Introduction

Economic development has a close mutual relationship with primary energy consumption (PEC) of natural resources. As a relic, and fossil vitality is a vital inorganic resource for industrialization and population. And the main pouring force for progression for a developed economy [[1](#_ENREF_1)]. The role of China is an actual picture of perennial concern at international climate change negotiations, energy consumption, industries, and ruralization. By underscoring the win-win strategies, these efforts and commitment are dubious to China’s economic development, and China’s high leveraged at climate change [[2-4](#_ENREF_2)]. Climate change resulting from anthropogenic greenhouse gas (GHG), energy consumption, industrialization, and value-added service fundamental challenges for societies [[3](#_ENREF_3)]. As a developing economy, China influences the environment, and its new industrial growth is making a new challenge for the rest of the world. A increasing effect on the resource of energy demand and supply of the world resources market paid attention to China [[5](applewebdata://6D1B99DE-14E7-47F5-89D4-97D5FED72B59#_ENREF_5)]. Rapid urbanization and value-added industrialization show environmental challenges [[6](#_ENREF_6), [7](#_ENREF_7)].

By the stochastic method, this study analyzed energy consumption, electricity generation, value-added industries, and ruralization. The Beta Decoupling Technique (BDT) is using to examine the individual effects of indicators. IPAT techniques assessing potential action, and re-conceptualize the identity of IPAT, remaining its impact(log). By disaggregating T into consumption per unit of GDP (C) and consumption per unit impact (T), it identifies like I=PACT [[8](#_ENREF_8)].The traditional concept of IPAT identity is analyzing the CO2 emissions, so the total emissions show that (I), population (P), per capita GDP (A), and CO2 emissions per unit of GDP (T) [[9](#_ENREF_9)]. IPAT planned into a stochastic model, calling it STIRPAT for stochastic influence by regression on population, affluence, and technology. The stochastic model (STIRPAT) has used to measure the effects of driving forces on a variety of environmental change by CO2 emissions [[10-12](#_ENREF_10)].

## 1.2. Primary energy consumption

[[13-15](#_ENREF_13)]Illustrated that China’s energy consumption increase continuously after 2014-2015, it recorded at 0.017%, and up to 2017-2018, it estimated 0.04% (show in Fig-1). The primary energy consumption is analyzing with oil, natural gas, coal, nuclear, hydroelectricity, and renewable energy consumption.

*Figure 1: % change in Primary energy consumption*

Therefore, in this context of primary energy consumption, China’s economic growth and energy consumption by taking the sanctuary of resources as the breakthrough point aim not only to provide a sufficient theoretical basis for China but also to articulate future energy development plan in the world.

## 1.3. Electricity generation

[[16](#_ENREF_16)] illustrated that electricity generation in China increased by 19.67% ifrom 2017 to 2020, 41.01% in 2020-2035, and expected to rise more 13.05% in 2035-2050. In 2050, the total electricity generation in China estimates to amount to 15,324 TWh. [[17](#_ENREF_17)] shows that tremendous economic activities affect China, and it is the most vital contribution to increase CO2 emissions from electricity generation, and efficiency influence plays the dwarf role in reducing CO2 emissions.

Figure 2: % change in CO2 emissions and electricity generation production

Fig 2 showed a % change in CO2 emissions because of electricity generation. A change in CO2 emission recorded the cause of gas and coal consumption from 2003 to 2005. As per the high demand for electricity consumption, China produces electricity by other natural resources and controls the environmental effects of CO2 emissions.

## 1.4. Value-added industries

As value-added industries, China is the worlds’ largest emitter of a greenhouse, and it bears a grueling task of emission reduction (ER) and energy conservation (EC) from different primary, secondary, and territory industries. In 2015, the [National Bureau of Statistics of China](http://www.stats.gov.cn/english/pressrelease/201510/t20151019_1257742.html) illustrated that the investment in primary industries reached up to 3.8% (3,919.5 billion yuan), 6% (19,779.9 billion yuan) in secondary, and 8.4% (25,0779.9 billion yuan) recorded in territory industries.. because of the vast majority of industrial processes, CO2 arising from energy consumption and makes up greenhouse gas emissions in China.

The new strategies of China to reduce CO2 emissions up to 2030 from the key industries

[[18](#_ENREF_18), [19](#_ENREF_19)]. However, the process of industrialization has forced China to confront the dual burden of air pollution and greenhouse gas emissions [[20](#_ENREF_20)]. The cement is the most crucial for economic growth and surging urbanization [[21](#_ENREF_21)].

Henceforth because production, industrial growth is detrimental to the ecosystem, planet, and inhabitants.

Figure 3: Value-added industries (% change)

Fig 3 is showing a % change in CO2 emissions because of value-added industries and the highest CO2 emission recorded during 2009. China's industrial revolution has been started after 1993 and boost economic growth.

The model of this paper is proposed by Beta Decoupling Techniques (BDT), which is commonly used for the % change of the indicators and shows elasticity. In this paper, BDT techniques are expended for economic growth on energy consumption with the stochastic model (IPAT) and used in the discussion of the relationships among CO2 emissions, growth (GDP), primary energy consumption (PEC, OIC. NGC, COC, NUC, HCC, and RGGB), electricity generation (EGO, EGS, and EGC), value-added industries (VPI, VSI, and VATI), and population (UPG and RUL). This study examines indicators effects by BDT, where IPAT is showing four different clusters, and further changes of CO2 emissions have been analyzed by decoupling. However, the prior review that this research method still has paid insufficient attention to the decoupling of different energy sources by the effects of GDP, energy consumption, electricity generation, value-added industries, and population. Disregard regarding primary energy types may lead to differentiated decoupling in the analyzed China economy during 1989-2018.

Because of a high dependence on resources (fossil energy), China can achieve resource security cause of accelerating the transition to non-fossil. The relationship between CO2 emissions and energy consumption with economic growth can not only broaden measure energy sustainable but also lay a valuable practical foundation for the consciousness of China’s sustainability economic growth in the coming years. The prior research ignores the energy consumption with value-added industries, electricity production, and the ruralization effects with GDP [[22](#_ENREF_22), [23](#_ENREF_23)]. CO2 emissions in China do not cause of business industries, and the peak will increase at gigatonnes (Gt) in 2020. They ignore value-added industries, electricity production by coal, and the ruralization effect [[24](#_ENREF_24)]. The enormous pressure put on Chinese industries’ reason for CO2 emissions. It shows to reduce CO2 emissions by a different way to technological implementation and proper strategies [[25](#_ENREF_25)]. Besides, direct consumption of power and healing energies in primary and tertiary industries was lower than secondary industries but showing the increase of CO2 emissions, and it reduced by both sides of supply and demand afterward [[26](#_ENREF_26)].

The existing research examined the relationship between CO2 emissions, economic growth, energy consumption, electricity production, value-added industries, and population with stochastic methodfrom the production and development side. The stochastic method (STIRPAT), used to calculate the CO2 emissions from 1989 to 2018.

This paper discusses the BDT relationship between CO2 emissions change the cause of economic growth, energy consumption, electricity production, value-added industries, population, production and consumption side in China. This paper compares and analyses the decoupling situation and change in % from different perspectives of STIRPAT clusters.

## 2. Methodology

## 2.1. Beta decoupling model (BDM)

In this study, we select indicators according to IPAT clusters where I (CO2 emissions), P (URL and RUL), A (GDP), T (PEC, OIC. NGC, COC, NUC, HCC, RGGB, EGO, EGS, EGC, VPI, VSI, and VATI). These indicators are highly correlated and co-integrated in industries. The distribution of clusters is showing on the IPAT technique, where I indicated that CO2 emissions, P (population), A (GDP), and T (technology). Furthermore, technology is analyzing by GDP and population. This data is adjusted by covariance and variane for the computation of α and β.

The decoupling value based on individual change in CO2 emissions and how a 1% change effects population (P), growth (A), and technology (T). After fulfilling all conditions of clusters strategy and beta decoupling model, selected four clusters (IPAT) are show (Table-1).

## 2.2. STIRPAT model

At for resources and environmental level of primary energy consumption, the decoupling technique used. The decoupling techniques is to describe the relationship between economic growth, resources, environment, value-added industries, and population. However, the environment does not change with economic growth.

This study aims at investigating the decoupling of CO2 emissions for China. This study shows economic growth, primary energy consumption, electricity generation, value-added industries, population, and decoupling of CO2 emissions with the STIRPAT model. The decoupling factors expanded by IPAT and the influence measure by GDP. Table 1 shows indicators, , used for analysis, and a description and sources of data, are presented with different abbreviations. We use panel data to analyze China data-set from 1989-2018.

Table 1:Variables definition and codes

| **Variables** | **Code** | | **IPAT** | **Description** |
| --- | --- | --- | --- | --- |
| CO2 emissions | CO2 | | **I** | Carbon dioxide emissions (BP-data) |
| Urbanization level (10000 person) | UPG | | **P** | % of total population living in urban areas. |
| Ruralization (POP/1 million) | RUL | |  | The total population is represented by the de facto definition of population. It is estimated on the basis of the midyear value.  and midyear value. |
| Gross domestic product at constant price (100 million yuan) | GDP | | **A** | GDP is showing the purchaser’s prices, and it is the sum of gross value added by all resident. |
| Primary energy consumption (Mote) | PEC | T1 | **T** | Energy consumption is indicating the total energy demand in China. |
| Oil consumption Tones | OIC | T2 |  | It is the amount of energy released by the burning process in different sectors. |
| Natural gas consumption | NGC | T3 |  | Natural gas |
| Coal consumption | COC | T4 |  | Coal consumption in industries and generation of electricity |
| Nuclear energy consumption | NUC | T5 |  | Energy consumption and the generation of electricity |
| Hydroelectricity consumption | HCC | T6 |  | Hydro-electricity |
| Renewable Geothermal, Biomass & others | RGGB | T7 |  | Natural and organic material |
| Electricity generation from oil (Twh) | EGO | T8 |  | Generation of electricity by oil, gas and coal |
| Electricity generation by gas | EGS | T9 |  |  |
| Electricity generation by coal | EGC | T10 |  |  |
| Value-added primary industries | VPI | T11 |  | Value-added primary, secondary, and territory industries illustrated the Economics value that company adds to services and products before offering them to the customer, which can boost revenue and profit. |
| Value-added secondary industries | VSI | T12 |  |  |

Source: <http://www.stats.gov.cn/tjsj/ndsj/2018/indexeh.htm>

The BDT of energy consumption and economic growth on the generation of electricity and value-added industries are similar to those on the primary energy consumption side. The decoupling index is the ratio of a 1% change in CO2 emissions to a 1% change in GDP. It indicated that the decoupling condition with the elastic range. In the first stage, the indicators of IPAT examine by covariance, variance, alpha, and beta. In the second stage, indicators distribute into three-level, decoupling, negative decoupling, and coupling. The critical value of positive and negative decoupling is +1 to +2 and -1 to -2.

The coupling value stated that zero and the subdivision continue with different attitudes of decoupling. The positive attitude of decoupling indicated strong, weak, and recessive conditions. And the negative decoupling is indicated that weak, strong, and expansive negative conditions. Fig 4 showing the RUL, URL, and RGGB are showing strong decoupling from 2000 to 2001 and weak decoupling with EGC from 2017 to 2018.

Figure 4:Beta Decoupling of indicators

Since the rate of change in economic growth greater than zero, this paper contains only four decoupling states: strong-weak decoupling, expansive coupling, and expansive negative decoupling. The highest and lowest decoupling shows a % change in CO2 emissions.

The sturdiest positive attitude of VPI shows that lots of industries have an influence on globally, and rise the pollution level. Hence, RUL and GDP attitudes are indicated a highly negative and indirect effect on CO2 emissions by lack of technology during 2015-2020 Fig 5.

Figure 5: Strong positive and negative decoupling

## 2.3. Data source

The energy consumption data of China come from the China Statistical Yearbook [(National Bureau of Statistics, 2018)](http://www.stats.gov.cn/tjsj/ndsj/2018/indexeh.htm). The main energy sources consume by primary energy consumption in multiple industries in China. This study selected primary-energy consumption (OIC, NGC, COC, NUC, HCC, and RGGB), electric generation (EGO, EGS, and EGC), and value-added industries (VPI, VSI, and VATI) of China.

China's population is analyzed and examine by URL and RUL. This paper concedes Beta Decoupling Techniques (BDT) [[27](#_ENREF_27), [28](#_ENREF_28)]. The BDT consisted of decoupling mechanisms by an individual change in variation (years) covariance, variance, alpha, beta, and decoupling, were analyzed the actual change in CO2 emissions by GDP, primary energy consumption, electricity generation, value-added industries, and population. Also, the decoupling mechanism is steadily growing, but from 1989 to 2018, it deserted. The adjustment of beta decoupling recognizes between +1 to -2, where the dependent variable (CO2 emission) will increase and decrease.

However, if the decoupling value is less than -1, we reduce CO2 emissions effects by technological skills and make proper strategies for growth and development. This paper uses GDP to indicate economic development and its influence on CO2 emissions by primary energy consumption, generation of electricity, value-added industries, and population.

## 3. Results and discussion

In this analysis, the decoupling relationship between CO2 emissions by GDP, energy consumption, electricity production, value-added industries and population used for China and all the data obtained from the National Bureau of statistics during 1989-2018.

## 3.1. Gross development product (GDP)

The covariance of GDP indicated that a 1% change in GDP should get change 0.00063% change in CO2 emissions because the covariance of beta is 0.0039%. The proportion of covariance calculates by GDP and its percentage change in five years. The GDP change is 0.139% for the beta proportion and mostly changes by 0.0039% in CO2.

A 1% change in CO2 is examining at 0.081%, and the value of decoupling recorded is 0.054% with a positive attitude. The decoupling access by a 1% change in CO2 subtracted from the computed beta value. Therefore, we expected that the beta value changes 0.0039% by a positive attitude.

However, the reality has happened that 0.081% change in CO2, so the decoupling was 0.045, and in the next year -1.314. After the addition of both decoupling values, the expected result is -1.269. It should suggest that we should reduce the development or at least modified the industries by gross product

Every five years, we compute CO2 emissions by decoupling techniques. A % change in CO2 emissions is also estimated each year cause of development and technical revolution in industries. Besides, the decoupling value recorded above -2, which indicates the CO2 emissions have increased due to growth and development from 1989 to 1993. The estimated decoupling of GDP record as -6.982 in the five years (2017-2021). However, the CO2 emissions will increase continuously in the coming years with increasing GDP. Like this, 30 years’ calculation examines within every five years in GDP, energy consumption, generation of electricity, value-added service of industries, and population.

China “New Era” to ensure health, sustainable economic development, and efficiency of energy to reduce CO2 emission from China. The new strategic policies resolve the CO2 emissions with a dynamic revolution of techniques [[29](#_ENREF_29), [30](#_ENREF_30)].

China is resolving the CO2 emissions issues from urbanization, population, and industrialization. And reduce the energy consumption demand from industrialization [[29](" \l "_ENREF_29" \t "_blank)]. In 2020, CO2 emissions intensity reduce by 40-45% and expected to reduced 60-65% by 2030 [[31](#_ENREF_31)].

## 3.2. Energy Consumption

Energy consumption entailed with PEC, OIC, NGC, COC, NUC, HCC, and RGGB. The covariance of PEC indicated that a 1% change in PEC should get change a 0.00023% change in CO2 emissions because the covariance of beta is 1.0524%. The proportion of covariance calculated by PEC and its percentage change in five years. The PEC change is 0.0765% as for the beta proportion. It mostly changes by 1.0524% in CO2. The 1% change in CO2 emissions records at 0.0765%, as the same reduction has recorded at 0.061% with a positive attitude.

The decoupling access by a 1% change in CO2 subtracted from the computed beta value. We expected that the beta value changes 1.0524% by a positive attitude. However, the reality has happened that 0.0765% change in CO2 emissions. Therefore, the decoupling was -1.003 and in the next year -1.123. The additive result of both decoupling value -2.126, it shows that reduction in PEC. Besides that, CO2 emissions are increasing, and we compute decoupling it for the next five years. The decoupling value is records form above -2, and it indicates that CO2 emissions increased cause of PEC from 1989 to 1993.

The expected decoupling of the PEC, OIC, NGC, COC, NUC, HCC and RGGB recorded -0.464, -0.604, -0.203, -0.742, -0.253, -0.269 and 0.042 in the period of 2017 to 2021. However, the CO2 emissions will increase with the consumption of PEC, OIC, NGC, COC, NUC, and HCC except for RGGB.

The resources of renewable energy of biomass decoupling show the positive attitude in every five periods from 1989 to 2018. However, the CO2 emission will reduce by renewable biomass. The primary energy of consumption (PEC, OIC, NGC, COC, NUC, HCC, and RGGB) with GDP has been plotted graphically in Fig 6.

The high-low line from 1999 to 2002 shows the highest positive attitude of RGGB with GDP, where the CO2 emission had reduced by the renewable biomass as for the alternative causes of energy. As well as NGS and OIC showed positive decoupling from 2003 to 2005 and 2011 to 2012. CO2 emissions reduce by oil and nuclear energy consumption.

The primary energy consumption quantifies and evaluates the energy or material metabolism and environmental loads of oil consumption through the establishment and use of petroleum flow and energy flow on the CO2 emissions [[32](#_ENREF_32)]. Currently, China has evacuated the second-largest petroleum processor and cause CO2 emissions, nearly a 60% increase in petroleum refining during 1990-2015 [[5](#_ENREF_5)].

In 2009, China's automobile industries and vehicle sale excess from U.S. vehicle sales to become the number one automobile in the world. It's relatively connected with the demand for consumption oil per vehicle fuel consumption rates, which will effectively increase oil demand and consumption and CO2 emissions by Chinese automobiles sectors [[33](#_ENREF_33), [34](#_ENREF_34)]. The combustion of biomass and fossil fuels contributes about 44% (range:36 to 78%) of the total global CO2 emissions [[35](#_ENREF_35)]. The consumption of natural gas has a significant negative impact on CO2 emissions, which indicates that a 1% change (increase) in natural gas will decrease CO2 emissions by 0.0549% [[36](#_ENREF_36)]. The advanced technologies make better policies and strategies for the utilization of energy, the use of appropriate and clean energy in China [[37](#_ENREF_37)].

Figure 6:GDP with Primary energy consumptions, energy generation, value added primary industries and Urban and rural population

China's dynamic strategies are improving continuously and the generation of coal structure for the reduction of CO2 emissions. Also, modified new techniques by using less-polluting and renewable (wind, water, solar, and nuclear) energies. However, nuclear energy sources are limited by public established impressions and modernization to utilize by the safe side. Biomass energy is also clean energy that can consistently use to achieve energy problems for the sustainability of an environment [[14](#_ENREF_14), [38](#_ENREF_38)].

## 3.3. Generation of Electricity

It elicits electricity generation with EGO, EGS, and EGC. It shows the covariance of the EGO that a 1% change in EGO should get a change of 0.00065% change in CO2 emissions because the covariance of beta is 0.0217%. The covariance of proportion calculates by EGO and its percentage change in five years. EGO change is 0.2301%, and as for the beta proportion, it most changes by 0.0217% in CO2. 1% change in CO2 recorded 0.2301%, so we have recorded some reduced value in decoupling is -0.2636% with a negative attitude. Decoupling access by a 1% change in CO2 subtracted from the computed beta value. Therefore, we expected that the beta value changes 0.0217% by a positive attitude.

However, the reality happened that 0.2301% change in CO2 emissions. The decoupling recorded 0.028, and in the next year with -0.046, the addition of both decoupling values is -0.018. It showed that CO2 emissions increased the cause of generation of electricity by oil, and as same we process as for each period.

The estimated decoupling value is above -2, so it shows that it increases CO2 emissions because of the high consumption of EGO from 1989 to 1993. Fig 6 showed that generation of electricity by oil, gas, and coal (EGO, EGS, and EGC) with GDP, where electricity produce by EGO shows high-low line intensity with a positive attitude and CO2 emission had reduced during 2011 to 2013. EGC shows a positive attitude of decoupling from 2017 to 2018, where the CO2 emission had reduced by EGC. In China, electricity generation is contingent on coal, which has led to an increase in CO2 emissions.

Because of high pressure, China cut its CO2 emissions from all sources of coal and announced it would cut CO2 emissions (per-unit) of GDP by 40% to 45% in 2020 from 2005[[17](#_ENREF_17), [39](#_ENREF_39), [40](#_ENREF_40)]. As for the reduction of CO2 emissions, environmental quality, water shift from coal to shale gas will improve consumption and public health [[41](#_ENREF_41), [42](#_ENREF_42)].

## 3.4. Value-added Services

The value-added services of the industries obtain by primary, secondary, and tertiary manufacturing industries with VPI, VSI, and VATI. It shows the covariance of VPI that a 1% change in VPI should get a change -0.000298% change in CO2 emissions because the covariance of beta is -0.3245%. The proportion of covariance calculated by VPI and its percentage change in five years. VPI change is 0%, as for the beta proportion, it most changes by -0.3245% in CO2. 1% change in CO2 recorded 0%, so we have recorded some increased value in decoupling is 0.0394% with a positive attitude. Decoupling access by a 1% change in CO2 subtracted from the computed beta value.

Therefore, we expected that beta value changes -0.3245% by a negative attitude. Estimated change in CO2 emissions by 0% and the decoupling recorded at 0.374, and in the next year with 0.785. The additive value of both decoupling records 1.159.

Figure 7: CO2 emission industrial sectors (million tons)

The expected decoupling value of the VPI, VSI, and VATI occurred as 0.104, 0.562, and -0.312 from 2017 to 2021. However, the CO2 emissions will increase from the value-added territory industries (VATI) and reduce by VPI and VSI. Value-added industries results show a positive attitude in one to six periods. However, CO2 emissions reduce by VPI. CO2 emissions have increased in the 5th and 6th periods of VSI and VATI.

The value-added industries of the primary, secondary, and territory (EGO, EGS, and EGC) with GDP plotted in Fig 6. A high and low line from 2014 to 2018 shows the highest positive attitude of VSI with GDP, where the CO2 emission had reduced by the value-added industries from the territory (VATI). And VPI showed a positive decoupling between 1991 and 1994. CO2 emission had reduced by energy generation from primary added industries. As a prior study, 80% of energy consumption has contributed to the industrial sectors of China's total emission since 2005 [[43](#_ENREF_43" \o "(NCCC), 2012 #1921)] In 2014, China overtook the USA as the world's biggest CO2 emitter [[44](#_ENREF_44)].The estimated value recorded that China (9,135 million tons), 177% of the USA emissions, and 28.21% of global emissions [[45](#_ENREF_45)].

Fig 7 showed that at the end of 2019, 1,423 (a million tons) have recorded by energy consumption by industrial sectors [46]. It's significant that cut CO2 emissions from energy consumption by 2030 goals [[46](#_ENREF_46)].

## 3.5. Urbanization and Ruralization

It elicits the urbanization and ruralization with URL and RUL. It shows the covariance of URL that a 1% change in URL should get change -1.4105e-05 change in CO2 emissions because the covariance of beta is -0.0967%. The proportion of covariance calculates by URL and its percentage change in five years.

The URL change is 0.0309%, and the beta proportion mostly changes by -0.0967% in the level of CO2 emissions. The 1% change in CO2 record 0.0309%, so we have recorded some reduced value in decoupling is 0.0300 with a positive attitude. The decoupling access by a 1% change in CO2 subtracted from the computed beta value. Therefore, we expected that beta value changes -0.0967%.

By the negative attitude, the reality happened that 0.0309% change in CO2 emissions, and the decoupling occurred 0.146. In the next year, increase with 0.979. Additive results show both decoupling is 1.125, and the estimated value is showing that CO2 emissions increase because of the URL. Same as that, we compute every five years CO2 emissions and show the increasing effect of URL from 1989 to 1993.

We recorded the expected decoupling of the URL and RUL 1.532 and -14.850 from 2017 to 2021. However, the CO2 emissions will increase from the ruralization (RUL) and reduce from urbanization (URL). Urbanization decoupling shows a positive attitude in 1,2,3 and 6 periods, where the CO2 emissions reduce by URL. CO2 emissions have increased in 4th and 5th periods in URL and 1st, 3rd, 5th and 6th periods of RUL Table 2. The urbanization and ruralization (URL and RUL) with GDP plotted in Fig 6.

The High-low line from 1997 to 2002 shows the highest positive attitude of RUL with GDP. In this stage, the value of CO2 emissions reduced by the ruralization of the population. The URL showed positive decoupling between 1997 and 2004. The CO2 emissions intensity reduces by urbanization.

As prior research, China contributed approx. 10% of global greenhouse gas emissions in 2017, where 71% of global CO2 emission contributed from the urban areas, because of high-tech and socioeconomic activities [[47](#_ENREF_47)].

The environment influences China's urbanization 14,000 Km2 areas experienced a high concentration of CO2 emissions [[48](#_ENREF_48)]. The rapid dimension in construction and renovation of space heating is obsessed by the urbanization [[49](#_ENREF_49)]. The higher intensity of CO2 emissions is recorded in the urban territories than residential or rural areas because of the higher volume of traffic and denser population [[50](#_ENREF_50), [51](#_ENREF_51)].

[[47](#_ENREF_47)].

Table 2: Decoupling of variables

Table 2 is showing the periods stand in five years and we compute decoupling for each period from 1989 to 2018. Arrow indications show a change in valuation, increase and decrease by yellow, light green, and red. However, over 1% change in decoupling is showing by light green, and less than -1% change is showing by purple. **Note:**Variable’s definition stated in**Table 1**

Table 3: Percentage change of indicators

**Note:** Variable’s definition indicated in **Table 1**

Table 3 is representing that 1% change in each period with the effect of a change in valuation, increase, and decrease by yellow light green, and red arrow. The light blue, yellow colors show the top and bottom changes by 10% in different periods. A 1% change in NUC (0.7248), HCC (0.3126), and RGB (0.5072) is showing a 1% change in CO2 emissions by positive decoupling of the NUC (0.049), HCC (0.3126), and RGB (0.5072) in the third and fourth periods.

Besides, that the pivot chart is showing a sum of decoupling with GDP effects, and it represents that a 1% change in CO2 emissions influence by decoupling from 1989 to 2018. We record the highest decoupling intensity on RUL. And showing that the percentage change in CO2. However, it might have effected by a tremendous level of urbanization to ruralization Fig 8.

Figure 8: Pivot chart with GDP

Table 4: Five years Beta

**Note:** Variable’s definition indicated in **Table 1**

The α and β results are impeaching foresee, and compare the estimated value of a 1% change in indicators, change in CO2 emissions because the covariance of β shows a positive or negative attitude. We calculate the proportion of covariance by each indicator and its percentage of change in five years in each period. Table 4 is comparing the five-year estimated results of all variables with the predicted value. A measure portfolio aptitude with a 1% change in GDP prefers to invest in each variable by higher α.The 1% change in CO2 emissions results from the α propensity, and the investor use β to determine how much downside expects by a difference in investment in indicators.

Evaluation of β analyzed by risk-averse on low and take the target on higher β hope to come for volatility. The URL (3.2863), PE (1.1054), and VSI (1.2481) show higher results, and their decoupling indicated that we should reduce CO2 emissions by PEC, VSI, and URL. Because a 1% change in indicators has to change in emission level and created volatility. Table 5 shows the predicted results of each period with top-bottom 10% results, where the 1% change in PEC, VSI, and URL regarding -0.005, 0.1289, and 0.2754, should get a change in CO2 emissions,

.

Table 5: Five years Alpha

**Note:** Variable’s definition indicated in **Table 1**

## 4. Conclusions and policy implications

In this study, we used the Beta Decoupling Techniques (BDT) to calculate the CO2 emissions by GDP, energy consumption (PEC, OIC, NGC, COC, NUC, HCC, and RGGB), electricity generation (EGO, EGS, and EGC), value-added industries (VPI, VSI, and VATI), and population (URL and RUL) of China from 1989 to 2018. We collect the relevant energy data set from the [National Bureau of Statistics of China](http://www.stats.gov.cn/english/) from energy consumption, industrialization, and population.

We analyzed the decoupling relationship between carbon emission, gross domestic product, primary energy consumption, electricity generation, value-added industries, and population of each period in China with the BDT. The top 10% results analyze the RGGB, EGO, EGS, VPI, VATI, URL, and RUL. Also, in the 3rd and 6th periods, the EGC and RUL estimated value is over 10% on the primary energy consumption, electricity generation, value-added industries, and population from 1989 to 2018. It shows a trend of increase with time. The EGC and RUL still accounted for a large proportion in the period 2017-2021, and over 90% of the total energy consumption.

The above result shows the energy consumption resources in China. From the eventual overall decoupling effect on energy consumption, electricity generation, and urbanization. We calculate the decoupling state based on the Beta Decoupling Techniques (BDT).

It analyzed the economic growth of energy consumption, industrial production, electricity generation, and population. China achieves a robust decoupling state and less dependent on renewable sources of vitality and fossil energy. Also, China has the countless ability to assurance resources security.

The decoupling significantly overestimated the energy level in China, especially on the industrial and ruralization side. The decoupling estimation of unique sources of energy concluded that the dependence of China’s rapid economy on a different level of energy sources differed from the perspective of energy consumption, electricity generation, and urbanization.

According to the above results, the decoupling in 2nd (NUC, RGGB, and EGS), 4th and 5th (EGO), 3rd 5th and 6th (RUL) periods state that less than 10% results of negative decoupling. The CO2 emissions will be increases in each period, so we should reduce the energy consumption electricity generation by oil, and ruralization. The illustrated results show that CO2 emissions are less dependent on COC, NUC, RGGB, EGO, EGS, VSI, and VSTI in a different period. Based on the above decisions, this paper situates forward relevant strategies recommendations.

First, the total composition of energy is vital issues that affect resource security and energy development strategies [[52](#_ENREF_52), [53](#_ENREF_53)]. It is most necessary to re-change growth and control by the level of energy. Besides, the population, solicitation of clean energy (solar, water, nuclear, wind, geothermal, tidal, and biomass), and to build the low carbon energy sources by technology. Also, develop a mechanism of renewable vitality aiming to improve the efficiency of utilization, energy conversation, energy transformation, and consumption [[49](#_ENREF_49), [54](#_ENREF_54)].

Second, each country needs to take serious action against CO2 emissions [[55](#_ENREF_55)]. Therefore, they can take measures to energy demand whenever planning to develop enormous industries and control emissions reduction measures with changing technology.

As for the development strategies, the government should expand its investment in the capital-intensive primary, secondary, and territory industries and promote the green technology emerging industries by enhancing technological innovations.

The focus of reducing the growth rate of energy consumption should be on improving energy efficiency use. Also, control and change strategies in natural-gas resources in China, how to secure and ensure the safe supply of natural gas should be the focus on tremendous macro policy.

Contemplating that the industrial structure, they relate it to GDP, the emphasis of future research ought to be on sightseeing the relationship between energy and economic growth from the perspective of primary energy consumption, electricity generation, industries, and population.

We recommend it to take optimization of demand and supply structure as the to provide cherished policy recommendation for adjusting China’s economic sustainability and enhancing economic development structure.

## Reference:

1. Zhang Z. Decoupling China’s Carbon Emissions Increase from Economic Growth: An Economic Analysis and Policy Implications. World Development. 2000;28(4):739-52. doi: <https://doi.org/10.1016/S0305-750X(99)00154-0>.

2. Ren S, Hu Z. Effects of decoupling of carbon dioxide emission by Chinese nonferrous metals industry. Energy Policy. 2012;43:407-14. doi: <https://doi.org/10.1016/j.enpol.2012.01.021>.

3. Schmidt TS, Schneider M, Hoffmann VH. Decarbonising the power sector via technological change – differing contributions from heterogeneous firms. Energy Policy. 2012;43:466-79. doi: <https://doi.org/10.1016/j.enpol.2012.01.041>.

4. Tasdoven H, Fiedler BA, Garayev V. Improving electricity efficiency in Turkey by addressing illegal electricity consumption: A governance approach. Energy Policy. 2012;43:226-34. doi: <https://doi.org/10.1016/j.enpol.2011.12.059>.

5. Wang H, Dai H, Dong L, Xie Y, Geng Y, Yue Q, et al. Co-benefit of carbon mitigation on resource use in China. Journal of Cleaner Production. 2018;174:1096-113. doi: <https://doi.org/10.1016/j.jclepro.2017.11.070>.

6. Zhou S, Wei W, Chen L, Zhang Z, Liu Z, Wang Y, et al. The impact of a coal-fired power plant shutdown campaign on heavy metals emissions in China. Environ Sci Technol. 2019. doi: <https://doi.org/10.1021/acs.est.9b04683>.

7. Zhao X, Zhang X, Li N, Shao S, Geng Y. Decoupling economic growth from carbon dioxide emissions in China: a sectoral factor decomposition analysis. . J CleanProd 2017;142:3500–16. doi: <https://doi.org/10.1016/j.jclepro.2016.10.117>.

8. Waggoner PE, Ausubel JH. A framework for

sustainability science: a renovated IPAT identity. . Proceedings of the National Academy of Sciences 2002;99 (12):7860 -5.

9. Dietz T, Rosa EA. Rethinking the environmental impacts of population, affluence and technology. . Human Ecology Review 1994;1:277 /300.

10. York R, Rosa EA, Dietz T. Footprints on the earth: the environmental consequences of modernity. . American Sociological Review 2003;68 (2):279 /300.

11. York R, Rosa EA, Dietz T. Bridging environmental science with environmental policy:Plasticity of population, affluence, and technology. . Social Science Quarterly 2002;83(1):18 -34.

12. Shi A. The impact of population pressure on global carbon dioxide emissions, 1975 /1996: evidence from pooled cross-country data. . Ecological Economics 2003;44(1):24 /42.

13. Li J, Wei W, Zhen W, Guo Y, Chen B. How green transition of energy system impacts China’s mercury emissions. . Earth’s Future 2019;7(1407–1416). doi: <https://doi.org/10.1029/2019EF001269>.

14. Yang J, Cai W, Ma M, Li L, Liu C, Ma X, et al. Driving forces of China’s CO2 emissions from energy consumption based on Kaya-LMDI methods. Science of The Total Environment. 2020;711:134569. doi: <https://doi.org/10.1016/j.scitotenv.2019.134569>.

15. Jie T, Wei WD, Jiang L. A sustainability-oriented optimal allocation strategy of sharing bicycles: evidence from ofo usage in Shanghai. . Resour Conserv Recycl. 2020;153. doi: <https://doi.org/10.1016/j.resconrec.2019.104510>.

16. Wong S. Total electricity generation forecast in China 2017-2050 China: Statista 2020. Available from: <https://www.statista.com/statistics/977374/china-total-electricity-generation-forecast/>.

17. Zhang M, Liu X, Wang W, Zhou M. Decomposition analysis of CO2 emissions from electricity generation in China. Energy Policy. 2013;52:159-65. doi: <https://doi.org/10.1016/j.enpol.2012.10.013>.

18. Xia F, Zhang X, Cai T, Wu S, Zhao D. Identification of key industries of industrial sector with energy-related CO2 emissions and analysis of their potential for energy conservation and emission reduction in Xinjiang, China. Science of The Total Environment. 2020;708:134587. doi: <https://doi.org/10.1016/j.scitotenv.2019.134587>.

19. Yu Y, Jin Z-x, Li J-z, Jia L. Low-carbon development path research on China’s power industry based on synergistic emission reduction between CO2 and air pollutants. Journal of Cleaner Production. 2020;275:123097. doi: <https://doi.org/10.1016/j.jclepro.2020.123097>.

20. Ofosu-Adarkwa J, Xie N, Javed SA. Forecasting CO2 emissions of China's cement industry using a hybrid Verhulst-GM(1,N) model and emissions' technical conversion. Renewable and Sustainable Energy Reviews. 2020;130:109945. doi: <https://doi.org/10.1016/j.rser.2020.109945>.

21. Chen J, Shen L, Shi Q, Hong J, Ochoa JJ. The effect of production structure on the total CO2 emissions intensity in the Chinese construction industry. Journal of Cleaner Production. 2019;213:1087-95. doi: <https://doi.org/10.1016/j.jclepro.2018.12.203>.

22. Muhammad B. Energy consumption, CO2 emissions and economic growth in developed, emerging and Middle East and North Africa countries. Energy. 2019;179:232-45. doi: <https://doi.org/10.1016/j.energy.2019.03.126>.

23. Baz K, Xu D, Ampofo GMK, Ali I, Khan I, Cheng J, et al. Energy consumption and economic growth nexus: New evidence from Pakistan using asymmetric analysis. Energy. 2019;189:116254. doi: <https://doi.org/10.1016/j.energy.2019.116254>.

24. Xu G, Schwarz P, Yang H. Adjusting energy consumption structure to achieve China's CO2 emissions peak. Renewable and Sustainable Energy Reviews. 2020;122:109737. doi: <https://doi.org/10.1016/j.rser.2020.109737>.

25. Lv Q, Liu H, Wang J, Liu H, Shang Y. Multiscale analysis on spatiotemporal dynamics of energy consumption CO2 emissions in China: Utilizing the integrated of DMSP-OLS and NPP-VIIRS nighttime light datasets. Science of The Total Environment. 2020;703:134394. doi: <https://doi.org/10.1016/j.scitotenv.2019.134394>.

26. Jiang T, Yang J, Huang S. Evolution and driving factors of CO2 emissions structure in China’s heating and power industries: The supply-side and demand-side dual perspectives. Journal of Cleaner Production. 2020;264:121507. doi: <https://doi.org/10.1016/j.jclepro.2020.121507>.

27. Xie P, Yang F, Mu Z, Gao S. Influencing factors of the decoupling relationship between CO2 emission and economic development in China’s power industry. Energy. 2020;209:118341. doi: <https://doi.org/10.1016/j.energy.2020.118341>.

28. Wang Q, Zhang F. The effects of trade openness on decoupling carbon emissions from economic growth – Evidence from 182 countries. Journal of Cleaner Production. 2021;279:123838. doi: <https://doi.org/10.1016/j.jclepro.2020.123838>.

29. Xie P, Gao S, Sun F. An analysis of the decoupling relationship between CO2 emission in power industry and GDP in China based on LMDI method. Journal of Cleaner Production. 2019;211:598-606. doi: <https://doi.org/10.1016/j.jclepro.2018.11.212>.

30. Yang L, Xia H, Zhang X, Yuan S. What matters for carbon emissions in regional sectors? A China study of extended STIRPAT model. Journal of Cleaner Production. 2018;180:595-602. doi: <https://doi.org/10.1016/j.jclepro.2018.01.116>.

31. M. Zhang YS. Exploring influence factors governing the changes in China’s final energy consumption under a new framework. Nat Hazards 2015;78 653-68.

32. Jia F-R, Jing W-T, Liu G-X, Yue Q, Wang H-M, Shi L. Paraffin-based crude oil refining process unit-level energy consumption and CO2 emissions in China. Journal of Cleaner Production. 2020;255:120347. doi: <https://doi.org/10.1016/j.jclepro.2020.120347>.

33. Wang M, Hong H, Johnson, L. H, D. Projection of Chinese Motor Vehicle Growth, Oil Demand, and CO2 Emissions through 2050. ANL/ESD/06-6. . Argonne National Laboratory, Argonne, IL, December.: 2006.

34. Wang Z, Jin Y, Wang M, Wei W. New fuel consumption standards for Chinese passenger vehicles and their effects on reductions of oil use and CO2 emissions of the Chinese passenger vehicle fleet. Energy Policy. 2010;38(9):5242-50. doi: <https://doi.org/10.1016/j.enpol.2010.05.012>.

35. Zhang J, Smith KR, Uma R, Ma Y, Kishore VVN, Lata K, et al. Carbon monoxide from cookstoves in developing countries: 1. Emission factors. Chemosphere - Global Change Science. 1999;1(1):353-66. doi: <https://doi.org/10.1016/S1465-9972(99)00004-5>.

36. Dong K, Sun R, Hochman G, Zeng X, Li H, Jiang H. Impact of natural gas consumption on CO2 emissions: Panel data evidence from China’s provinces. Journal of Cleaner Production. 2017;162:400-10. doi: <https://doi.org/10.1016/j.jclepro.2017.06.100>.

37. Muhammad B, Khan S. Effect of bilateral FDI, energy consumption, CO2 emission and capital on economic growth of Asia countries. Energy Reports. 2019;5:1305-15. doi: <https://doi.org/10.1016/j.egyr.2019.09.004>.

38. Wang Z. Does biomass energy consumption help to control environmental pollution? Evidence from BRICS countries. Sci Total Environ. 2019; 670 1075-83.

39. Kong Y, Khan R. To examine environmental pollution by economic growth and their impact in an environmental Kuznets curve (EKC) among developed and developing countries. PloS one. 2019;14(3). doi: <https://doi.org/10.1371/journal.pone.0209532>.

40. Khan R. Effects of Energy Consumption on GDP: New Evidence of 24 Countries on Their Natural Resources and Production of Electricity. Ekonomika. 2020;99(1):26-49. doi: <https://doi.org/10.15388/Ekon.2020.1.2>.

41. Berk I, Kasman A, Kılınç D. Towards a common renewable future: The System-GMM approach to assess the convergence in renewable energy consumption of EU countries. Energy Economics. 2018. doi: <https://doi.org/10.1016/j.eneco.2018.02.013>.

42. Jenner S, Lamadrid AJ. Shale gas vs. coal: Policy implications from environmental impact comparisons of shale gas, conventional gas, and coal on air, water, and land in the United States. Energy Policy. 2013;53:442-53. doi: <https://doi.org/10.1016/j.enpol.2012.11.010>.

43. (NCCC) NCCoCC. Second National Communication on Climate Change of The People's Republic of China

Beijing, China 2012.

44. IEA. CO2 emissions from fuel combustion, 2008, in: (IEA), I.E.A. (Ed.). Head of Communication and Information Office, Soregraph, France.: 2009.

45. IEA. CO2 emissions from fuel combustion 2018 Head of Communication and Information Office, Soregraph, France.: 2018.

46. EIA UEiA. Monthly Energy Review. USA: July 2020.

47. Zhang W, Cui Y, Wang J, Wang C, Streets DG. How does urbanization affect CO2 emissions of central heating systems in China? An assessment of natural gas transition policy based on nighttime light data. Journal of Cleaner Production. 2020;276:123188. doi: <https://doi.org/10.1016/j.jclepro.2020.123188>.

48. Fang C, Wang S, Li G. Changing urban forms and carbon dioxide emissions in China: A case study of 30 provincial capital cities. Applied Energy. 2015;158:519-31. doi: <https://doi.org/10.1016/j.apenergy.2015.08.095>.

49. Huo T, Li X, Cai W, Zuo J, Jia F, Wei H. Exploring the impact of urbanization on urban building carbon emissions in China: Evidence from a provincial panel data model. Sustainable Cities and Society. 2020;56:102068. doi: <https://doi.org/10.1016/j.scs.2020.102068>.

50. George K, Ziska LH, Bunce JA, Quebedeaux B. Elevated atmospheric CO2 concentration and temperature across an urban–rural transect. Atmospheric Environment. 2007;41(35):7654-65. doi: <https://doi.org/10.1016/j.atmosenv.2007.08.018>.

51. Gao Y, Lee X, Liu S, Hu N, Wei X, Hu C, et al. Spatiotemporal variability of the near-surface CO2 concentration across an industrial-urban-rural transect, Nanjing, China. Science of The Total Environment. 2018;631-632:1192-200. doi: <https://doi.org/10.1016/j.scitotenv.2018.03.126>.

52. Li J, Wei W, Zhen W, Guo Y, Chen B. How green transition of energy system impacts China’s mercury emissions. . Earth’s Future 2019;7:1407–16. doi: <https://doi.org/10.1029/2019EF001269>.

53. Aydin M. The effect of biomass energy consumption on economic growth in BRICS countries: A country-specific panel data analysis. Renewable Energy. 2019;138:620-7. doi: <https://doi.org/10.1016/j.renene.2019.02.001>.

54. Cheng C, Ren X, Wang Z, Yan C. Heterogeneous impacts of renewable energy and environmental patents on CO2 emission - Evidence from the BRIICS. Science of The Total Environment. 2019;668:1328-38. doi: <https://doi.org/10.1016/j.scitotenv.2019.02.063>.

55. Acheampong AO. Economic growth, CO2 emissions and energy consumption: What causes what and where? Energy Economics. 2018;74:677-92. doi: <https://doi.org/10.1016/j.eneco.2018.07.022>.
