# Supplementary material for: Beta decoupling relationship between CO2 emissions by GDP, energy consumption, electricity production, value-added industries, and population in China
Source: PLoS One. 2021 Apr 1;16(4):e0249444. doi: 10.1371/journal.pone.0249444 (PMC8016343; doi:10.1371/journal.pone.0249444)
Supplement: S2 File — (DOCX) [file pone.0249444.s002.docx]

Emmanuel Yamoah Cobbold

School of Finance and Economics

Jiangsu University

The People’s Republic of China (PRC).

[eycobbold@yahoo.com](mailto:eycobbold@yahoo.com)

**Introduction**

I am a final year PhD Degree student at the School of Finance and Economics at Jiangsu University, Jiangsu University, China. Majoring in Applied Economics, my research fields include; International Trade and Finance as well as Energy and Development Economics.

I also have vast experience of work in research publications technical writing skills. This we believe will enable policymakers to have a broader view of what other factors influence emissions aside the frequently researched.

Philosophy

Today’s academic and corporate worlds are not so much of a disjoint set. The two worlds look forward to having substantive information from researchers. The educational setup is expected to train and also bring vital findings through research. These findings will then be processed and implemented jointly with the corporate world. For instance, academics research into technological changes whilst industry players put these research findings into play. This will lead to the changes we all expect to see in our daily lives.

The onus, therefore lies on the educational sector; researchers in particular, to delve and conduct extensively and provide findings which when implemented will cause a positive change to society.

Conclusion

I look forward to conducting extensive researches and become a solid source of knowledge in Economics and Finance. My plans for the future also include mentoring young researchers and sharing my knowledge with the world through every means possible.

Emmanuel Yamoah Cobbold

[eycobbold@yahoo.com](mailto:eycobbold@yahoo.com)

18652831182
