# Supplementary material for: Beta decoupling relationship between CO2 emissions by GDP, energy consumption, electricity production, value-added industries, and population in China
Source: PLoS One. 2021 Apr 1;16(4):e0249444. doi: 10.1371/journal.pone.0249444 (PMC8016343; doi:10.1371/journal.pone.0249444)
Supplement: Attachment — Submitted filename: BDT-Response to Reviewers.docx [file pone.0249444.s004.docx]

**Authors' Response to Reviewers' Comments**

No: [PONE-D-20-34342]

Title: Beta decoupling relationship between CO2 emissions by GDP, energy consumption, electricity production, value-added industries, and population in China

Dear editor and reviewers,

Thank you very much for your letter and reviewers’ valuable suggestions on our manuscript entitled “*Beta decoupling relationship between CO2 emissions by GDP, energy consumption, electricity production, value-added industries, and population in China”* No: [PONE-D-20-34342]. Those comments are very valuable and helpful for improving our paper, as well as providing the important guiding significance to our research. According to the editor’s and reviewers’ comments, we have carefully checked and improved the manuscript. The questions in the comments were marked in blue, and the corresponding responses are listed below point by point.

**Response to additional requirements.**

1. Please ensure that your manuscript meets PLOS ONE's style requirements, including those for file naming. The PLOS ONE style templates can be found at

https://journals.plos.org/plosone/s/file?id=wjVg/PLOSOne_formatting_sample_main_body.pdf andhttps://journals.plos.org/plosone/s/file?id=ba62/PLOSOne_formatting_sample_title_authors_affiliations.pdf

**Reply:** Thank you very much for your valuable comments and suggestions. According to your valued comments, we have revised it carefully as follows.

We have revised the paper point by point by the valuable comment.

According to the Plos One manuscript body formatting guideline, I modified the whole paper, including heading, figures, and table style. The complex structure of Beta decoupling techniques (BDT) tables 2, 3,4, and 5 do not fulfill the requirement of table style, therefore as per given instruction all the tables are converted into figure caption like 6,9,11, and 12.

2. Thank you for stating the following in the Acknowledgments Section of your manuscript: "I acknowledge the helpful instruction and comments from my supervisor and thanks to the financial support of the National Natural Science Foundation of China of the Fujian University of Technology." We note that you have provided funding information that is not currently declared in your Funding Statement. However, funding information should not appear in the Acknowledgments section or other areas of your manuscript. We will only publish funding information present in the Funding Statement section of the online submission form. Please remove any funding-related text from the manuscript and let us know how you would like to update your Funding Statement. Currently, your Funding Statement reads as follows:

"Yes."

Please include your amended statements within your cover letter; we will change the online submission form on your behalf.

**Reply:** Thank you very much for your valuable comments and suggestions. According to your valued comments, we have revised it carefully as follows.

We have revised the paper point by point by the valuable comment.

As per the given instruction, I have excluded the funding information from acknowledgment (Line 28).

4. Please include a caption for figure 8.

**Reply:** Thank you very much for your valuable comments and suggestions. According to your valued comments, we have revised it carefully as follows.

We have revised the paper point by point by the valuable comment.

According to the Plos One manuscript body formatting guideline, I modified the figures and table captions under the stated requirements. Tables exported as TIFF figure, all the figure captions have been revised and cite and re-label. Figure 8 caption is updated now it shows figure 10.

**Reviewers' comments:**

**5.Reviewer's Responses to Questions**

Reviewer #1: 1 The abstract can be further refined.

**Reply:** Thank you very much for your valuable comments and suggestions. According to your valued comments, we have revised it carefully as follows.

We have revised the paper point by point by the valuable comment.

As per the given instruction, I have modified and refined the abstract and make it easy for the reader. likewise

"The sources of fossil energy are a vital cause of economic growth and influence on security, whereas energy enhances or promotes socio-economic stability and the environment. As the fossil energy sources supply has become progressively stern, reconnoitering the beta decoupling relationships between CO2 emissions, GDP, energy consumption, electricity consumption, value-added industries, and population in China, and the result will be favorable for illustrative the security of the resources. This study adopts the extended stochastic model (STIRPAT) with Beta Decoupling Techniques (BDT). This technique employs the decoupling situation by the alpha and beta effects from 1989 to 2018 and calculates the % change in CO2 emissions by GDP growth and energy consumption. The estimated results are negative and economic growth depends on coal and natural gas. First CO2 emissions annually increasing cause of rapid growth, energy consumption, and electricity production, and the structural contradiction of energy remained static. Second, the Value-added industries estimated that CO2 emissions reduce by primary industries. Third, the decoupling states of CO2 emissions and population show an inverse relationship. This paper suggests that China is sustainable and strengthen energy output, transmute the energy consumption structure, and advance development policies under the environmental circumstance."

2 There are more than five keywords in the abstract.

**Reply:** Thank you very much for your valuable comments and suggestions. According to your valued comments, we have revised it carefully as follows.

We have revised the paper point by point by the valuable comment.

As per the given instruction, I have used only the five most relevant and authentic words as keywords. Likewise, the Beta Decoupling Technique, stochastic model (STIRPAT), energy consumption, electricity production, and value-added industries.

3 The introduction does not elaborate on the topic of this article and its value, and we recommend

rewriting.

**Reply:** Thank you very much for your valuable comments and suggestions. According to your valued comments, we have revised it carefully as follows.

We have revised the paper point by point by the valuable comment.

As per the given instruction, the first section introduction is modified and improved with new research findings. Modified article lines are stated below.

Modified lines with track change:

1. Introduction: (237 to 253), (257 to 263), (266 to 270), (273 to 278), (319 to 353)

1.2 Primary energy consumption: (360 to 364)

1.3 Electricity generation: (386 to 391)

1.4 Value-added industries: (240 to 248)

4 The contribution of this article is not very obvious, and it is recommended to rewrite.

**Reply:** Thank you very much for your valuable comments and suggestions. According to your valued comments, we have revised it carefully as follows.

We have revised the paper point by point by the valuable comment.

As per the given instruction, the contribution of the article rewrites from the line 319 to 353.

A significant contribution of this study to the existing research is the Beta decoupling technique (BDT). (1) Mostly, existing studies sought causes for decoupling economic upheaval from CO2 emissions based on technological changes and production. (2) It used the decomposition approach because of the logarithmic mean division index (LMDI), which shows the index decomposition method. It limits the LMDI approach in case of technical efficiency and growth [24]. Therefore, it’s needed to introduce a more meaningful technique to determine the significant effects of CO2 emissions by growth and energy consumption. (3) The Beta decoupling technique expanded by planned (IPAT) into a stochastic model (STIRPAT) analysis of CO2 emissions. (4) We examined the decoupling among CO2 emissions, GDP, energy consumption, electricity production, value-added industries, and population for China from 1989 to 2018, while most prior studies have explored erstwhile to 1989. This research study should specify a thoughtful sound of Beta decoupling and its causes for the latest eras in China.

5 In the part 3, the reasons for the research results need to be explained and demonstrated.

**Reply:** Thank you very much for your valuable comments and suggestions. According to your valued comments, we have revised it carefully as follows.

We have revised the paper point by point by the valuable comment.

**As per the given instructions, the third part of the article amends and modified. The research results are explained and demonstrated line by line.**

Modified lines with track change:

## 3.1 Gross development product (GDP): **(740 to 750), and (757 to 774)**

## 3.2 Energy Consumption: (793 to 796), (833 to 840), (844 to 845)

## 3.3 Generation of Electricity: (916 to 942)

## 3.4 Value-added Services: (1002 to 1008), (1024 to 1090)

## 3.5 Urbanization and Ruralization: (1110 to 1118)

**Thank you very much again for your valuable comments and suggestions.**
