# Supplementary material for: Beta decoupling relationship between CO2 emissions by GDP, energy consumption, electricity production, value-added industries, and population in China
Source: PLoS One. 2021 Apr 1;16(4):e0249444. doi: 10.1371/journal.pone.0249444 (PMC8016343; doi:10.1371/journal.pone.0249444)
Supplement: Attachment — Submitted filename: BDT(2)-Response to Reviewers.docx [file pone.0249444.s005.docx]

**Authors' Response to Reviewers' Comments**

No: PONE-D-20-34342R1

Title: Beta decoupling relationship between CO2 emissions by GDP, energy consumption, electricity production, value-added industries, and population in China

Dear editor and reviewers,

Thank you very much for your letter and reviewers' valuable suggestions on our manuscript entitled *"Beta decoupling relationship between CO2 emissions by GDP, energy consumption, electricity production, value-added industries, and population in China”.* No: [PONE-D-20-34342R1]. These comments are very esteemed and helpful for meaningfully improving our paper. It is providing the imperative guiding significance to our research. According to the editors' and reviewers’ comments, we have carefully checked and improved the manuscript. The questions in the comments were marked in blue, and the corresponding responses are listed below point by point.

**Reviewers' comments:**

**Response to additional requirements.**

6. Review Comments to the Author

Reviewer #2: After a careful assessment of the manuscript, I believe this informative study is suitable for publication.

This article describes that the sources of fossil energy are a vital cause of economic growth and influence on security, whereas energy enhances or promotes socio-economic stability and the environment………. energy consumption.

Abstract and Introduction improvement: I happy to evaluate this interesting study. In my opinion, I have some guidelines for the authors to enhance the study quality before endorsing it for publication. As the Abstract is the main door or "FACE" of the manuscript, it should briefly present high-quality English with new information. I have suggested some studies to check the abstracts, improve yours, cite them in the introduction, and build your study objectives like these studies.

**Reply:** Thank you very much for your valuable comments and suggestions. According to your valued comments, we have revised it carefully as follows.

We have revised the paper point by point by the valuable comment.

Under your kindful consideration, I have changed sentences with pure wording and increase reader interest. I have also read your research papers, and after profound review, I modified the abstract and introduction portion. Likewise,

The credible sources of fossil energy efficiently are a vital cause of economic growth and considerable influence on adequate security. Whereas radiant energy positively enhances or ostensibly promotes socio-economic stability and the controlled environment. The fossil energy sources supply has become progressively stern in China and reconnoitering the beta decoupling relationships between CO2 emissions, GDP, energy consumption, electricity consumption, value-added industries, and population.

In the introduction portion, I have cited your research papers. As per the given valuable suggestion of your three research papers, I modified the 2^nd^ paragraph lines (112-134) and included the valued finding of your research with technical skills. Furthermore, as per the given instruction research manuscript carefully check the theoretical. The whole paper screening again lines to the line under technical and academic writing style. And progressively improve the advised reader's economic interest. Graciously according to manuscript objectives, all the cleared tables, figures and values verify again with the proper interpretation and clear the determined objectives with technical skills. In addition, as per the given instruction, the theoretical check and modified the significant contribution from the line 253 to 265.

Methods and Results

The results section of the paper presents a good view of the study. This work presents a notable investigation on a selected topic. I suggest including some graphical presentations to improve the quality of this study. Please see the proposed studies and see the graphical representation. Improve your work like these studies and cite them in this section.

**Reply:** Thank you very much for your valuable comments and suggestions. According to your valued comments, we have revised it carefully as follows.

We have revised the paper point by point by the valuable comment.

As per your valuable suggestion, I have already included three figures (1,2 and 3) lines (287, 317, and 363) in the introduction portion in the form of primary energy consumption, electricity production, and value industries.

The second portion (methodology) included two figures (4 and 5) lines (502 and 525) in the form of the Beta decoupling and a strong positive/negative attitude of decoupling. The third portion (result and discussion) included three Figures (7,8 and 10) lines (720,807, and 958) in the form of GDP with Primary energy consumptions, energy generation, value-added primary industries, and Urban and rural population. CO2 emission industrial sectors (million tons) and the Pivot chart with GDP. In addition, individually interpreted all the eight figures in lines 269, 319, 365, 484, 508, 676,816, and 941.

In this portion, I have also cited your three research papers and modified the separate sentences as for the valuable interest.

Conclusion
I suggest you make a separate heading of the conclusion and do not mix it with implications.

Policy Recommendations implication I again recommend you to make a separate heading of the Policy Recommendations.

The conclusion section is acceptable. Overall, this presents a good piece of research work. I recommend that authors do a little more work and revise this article accordingly. I suggest the authors check English quality and fix some weak sentences. If you have already taken English editing service, ask them to recheck the quality to meet scientific merit for publication. I endorse this manuscript for publication after minor corrections, as suggested.

**Reply:** Thank you very much for your valuable comments and suggestions. According to your valued comments, we have revised it carefully as follows.

We have revised the paper point by point by the valuable comment.

As per the given instruction, I have separated both portion recommendation (line 1056) and implication also theoretical check it as per reader interest.

Furthermore, the quality of English has been improved with editing services, and recheck the quality merit for publication.

Reviewer #3: Dear Authors,

Thank you for the opportunity for me to go through and read your Paper Titled: Beta decoupling relationship between CO2 emissions by GDP, energy consumption, electricity production, value-added industries, and population in China.

The authors have done a great a job by revising the paper. The Abstract and Introduction has been revised Accordingly. Also, the Research Results is well explained and Demonstrated, therefore the recommend this paper for Publication.

**Reply:** Thank you very much for your valuable comments and suggestions. According to your valued comments, we have revised it carefully as follows.

We have revised the paper point by point by the valuable comment.

Thank you so much for your kind compliments.

**Thank you very much again for your valuable comments and suggestions.**
